# The p53-PA200 Feedback Loop Modulates Age-Dependent Tumorigenesis

**DOI:** 10.3390/ijms27198783

**Published:** 2026-09-30

**Authors:** Chen-Yang Jiang, Yan-Li Wang, Nan Lv, Bing-Kun Cai, Tian-Xia Jiang

**Affiliations:** Ministry of Education Key Laboratory of Cell Proliferation & Regulation Biology, College of Life Sciences, Beijing Normal University, 19 Xinjiekouwai Avenue, Beijing 100875, China

**Keywords:** Proteasome activator PA200, p53, β-catenin, tumorigenesis, aging

## Abstract

Tumor incidence increases markedly with advancing age, yet the molecular mechanisms linking aging-driven senescence to tumorigenesis remain incompletely understood. The proteasome activator PA200 preserves genomic stability by mediating the degradation of acetylated histones during aging. However, its function in age-associated tumor progression is poorly defined. Here, we demonstrate that PA200 promotes aging-associated tumorigenesis independent of its histone-degrading activity. Elevated PA200 correlates with poor prognosis and advancing age in human lung cancer. PA200-deficient tumor cells exhibit suppressed proliferation and migration, accompanied by aggravated oxidative-stress-triggered cellular senescence. Mechanistically, p53 directly binds and represses *PSME4* transcription at its promoter. Reciprocally, PA200 facilitates proteasomal degradation of β-catenin, thereby sustaining p53 activity and forming a negative feedback loop. In the urethane-induced mouse lung tumor model, PA200 ablation markedly reduces tumor burden, especially in middle-aged mice. This effect is coupled with β-catenin accumulation, lowered p53 levels, and partial remodeling of the pro-inflammatory tumor microenvironment. Our findings uncover the p53-PA200-β-catenin feedback axis as a key driver of age-dependent lung tumorigenesis and highlight PA200 as a potential therapeutic target for elderly patients with lung cancer.

## 1. Introduction

Cancer rates increase significantly with advancing age, particularly for lung cancer, the incidence of which gradually increases with age, peaking among middle-aged and older adults between 50 and 75 years of age [1]. Understanding the link between tumorigenesis and cellular senescence is essential for the effective targeting of highly age-dependent malignancies. Aging and cancer are interconnected through underlying mechanisms, including endogenous and lifestyle factors, as well as via bidirectional crosstalk. This interplay renders old age not only a risk factor for cancer but also a critical parameter that must be considered for therapeutic decisions [2].

As organisms age, cumulative cellular stress, genomic instability, and the accumulation of senescent cells create a proinflammatory microenvironment that actively promotes cancer development and progression [3,4]. Specifically, senescent cells release a complex cocktail of inflammatory mediators, extracellular matrix-modifying proteins, and growth factors, collectively known as the senescence-associated secretory phenotype (SASP) [5,6,7,8,9]. This SASP remodels the tumor microenvironment (TME), enabling tumors to evade immune destruction and actively modulating tumorigenesis [10]. Furthermore, SASP-derived cytokines such as IL-1 [11], IL-6 [12], and TNF-α [13,14] can drive chronic inflammation and induce senescence in normal cells. Concurrently, this chronic inflammation accelerates immune cell aging, weakens immune function, and impairs the clearance of senescent cells and inflammatory mediators, thereby creating a self-reinforcing cycle between aging and inflammation [15]. Building on these protumorigenic functions of senescence, a two-step therapeutic paradigm termed the “one-two punch” has been developed. Tumor cells are first driven into senescence, followed by selective clearance via senolytic agents. Although this strategy yields encouraging results in gastric [16] and liver [17,18] cancer models, its effectiveness in lung cancer is not fully validated. It is therefore critical to define core regulators of senescence and their roles in lung tumorigenesis.

While multiple mechanisms link aging and cancer, dysregulated protein degradation pathways play a fundamental role in shaping protumorigenic SASP and promoting age-related tumorigenesis. These pathways control the stability of key oncogenic and senescence-modulating factors. Proteasome activity declines with advancing age, leading to accumulation of misfolded or aggregated proteins and gradually destabilizing protein homeostasis [19,20]. Efficient degradation of substrate proteins relies on proteasome-activating factors to modulate the activity of the 20S core particle [21,22,23,24]. The proteasome activator subunit PA200 (PSME4) binds to the 20S core particle proteasome to form the PA200 proteasome [22,25]. It specifically degrades acetylated core histones, among which acetylated H4K16 is notably upregulated during cellular senescence [26], somatic DNA damage responses and spermatogenesis [25]. Our previous studies have demonstrated that PA200 expression decreases during the aging process and that its deletion dramatically alters the deposition of active transcriptional hallmarks (H3K4me3 and H3K56ac) as well as transcription, thereby accelerating cellular and individual aging in mice, including immune malfunction and anxiety-like behavior, and shortening lifespan [27,28]. Further evidence has validated this polyubiquitin-independent degradation of H4 by PA200 in PRMT1-driven senescence, supporting PA200 as a potential senescence biomarker [28]. Moreover, PA200 has been reported to remodel proteasome function and diminish antigenic diversity, which compromises anti-tumor immunity in non-small cell lung cancer [29]. Given these dual biological functions, PA200 represents an attractive entry point for dissecting the molecular basis linking aging and tumorigenesis.

In this study, we report that the promoting effect of PA200 on tumorigenesis is more pronounced in aged individuals. We present evidence that PA200 drives age-dependent tumorigenesis by slowing tumor cell senescence, enhancing migration and invasion, and driving the expression of SASP components. Furthermore, we identify the potential molecular mechanisms underlying the maintenance of PA200 homeostasis via the β-catenin (CTNNB1) -mediated p53-PA200 feedback loop. This may provide an important insight into aging-driven anticancer therapies.

## 2. Results

### 2.1. PA200 Strongly Promotes Tumorigenesis in H_2_O_2_-Induced Senescent Tumor Cells

Our previous work demonstrated that PA200 is a critical factor for maintaining cellular youthfulness through the preservation of genomic stability [27]. Whether PA200 promotes aging-dependent tumorigenesis remains unclear. To address this question, we first analyzed *PSME4* transcription levels across multiple tumor types using the GEPIA database. This integrates The Cancer Genome Atlas (TCGA) tumor/adjacent-normal samples and GTEx healthy tissues to facilitate reliable tumor-normal comparisons. Our analysis identified markedly elevated *PSME4* expression in lung squamous cell carcinoma (LUSC), diffuse large B-cell lymphoma (DLBC), glioblastoma multiforme (GBM), and pancreatic adenocarcinoma (PAAD) (Figure 1a). Furthermore, interrogation of the TCGA database revealed that high PA200 expression correlates with unfavorable overall survival in patients with LUAD, adrenocortical carcinoma (ACC), low-grade glioma (LGG), and mesothelioma (MESO) (Figure 1b), suggesting a potential oncogenic role of PA200 in tumorigenesis and progression. Surprisingly, *PSME4* expression in tumor tissues of lung cancer patients ranging from 54 to 82 years was strongly positively correlated with patient age, with pronounced differences detected between the 68–82 years group and the 54–59 years group (Figure 1c). Collectively, these findings indicated that PA200 might promote tumorigenesis and tumor progression, especially in aged individuals.

To validate this hypothesis, we established wild-type (WT) and PA200-knockout (KO) mouse models via axillary inoculation of Lewis lung carcinoma (LLC) cells. Tumor volumes were significantly smaller in PA200-deficient mice compared with WT mice (Figure S1a,b). To further assess the effect of PA200 on cellular growth, we carried out clonogenic and proliferation assays using WT and PA200 KO tumor cells. The results showed that PA200 deficiency markedly suppressed cell proliferation (Figure S1c–e), as well as cell migration evidenced by scratch wound-healing assays (Figure S1f) and serum-stimulated migration assay (Figure S1g). Thus, the oncogenic activity of PA200 is largely driven by promoting tumor cell proliferation and migration.

Natural aging posits that aging is driven by cumulative oxidative damage [30]. In our cellular system, H_2_O_2_ treatment was used to accelerate the acquisition of senescent features. To determine whether PA200 plays a vital role in oxidative-stress-induced senescent tumor cells, we established a hydrogen peroxide (H_2_O_2_)-induced cellular senescence model to assess the effects of PA200 on proliferation and migration in senescent cells. At H_2_O_2_ concentrations ranging from 0 to 800 μM, the expression level of the senescence marker p16 significantly increased in a concentration-dependent manner (Figure 2a), confirming the successful establishment of the senescence model. Concurrently, PA200 expression gradually decreased as senescence progressed (Figure 2a). Regardless of H_2_O_2_ induction, compared with WT cells, PA200-deficient tumor cells exhibited significantly higher senescence levels, and this effect was more pronounced in the presence of H_2_O_2_ (Figure 2b,c), indicating an antagonistic relationship between PA200 and senescence. Additionally, PA200 deficiency significantly suppressed cell migration, with this inhibitory effect more prominent under senescent conditions (Figure 2d,e). It further exacerbated proliferative defects under senescence, as validated by a cell proliferation assay (Figure 2f). Notably, in untreated tumor cells, PA200 KO resulted in only a mild reduction in the proportion of cells in G1/S phase and a slight increase in the G2 phase population, indicating a relatively moderate inhibitory effect of PA200 depletion on cell cycle progression. In contrast, in the H_2_O_2_-induced oxidative stress model of senescence, the WT-H_2_O_2_ group already exhibited characteristic G2/M phase arrest, as reflected by an increased percentage of cells in G2 phase and a concomitant decrease in the G1/S phase population. Under oxidative stress, PA200 deletion significantly exacerbated this cell cycle arrest, as evidenced by the proportion of cells in G1/S phase reaching its lowest level, while the proportion of cells in the G2 phase fraction peaked. Further comparison between KO-H_2_O_2_ and KO cells revealed that oxidative stress treatment substantially aggravated G2 phase arrest in PA200-deficient cells, rendering them less capable of progressing through mitosis and severely impairing their proliferative capacity (Figure 2g). Collectively, these data indicated that the regulatory effect of PA200 on tumor cell proliferation was substantially amplified under senescent or stressed conditions. Specifically, PA200 effectively alleviated oxidative stress-induced G2/M phase arrest, thereby preserving the proliferative potential of senescent tumor cells. Thus, the impact of PA200 on the proliferative and migratory capacities of tumor cells was substantially amplified in senescent tumor cells, highlighting its prominent role in promoting tumorigenesis in aged tumor cells.

### 2.2. p53 Directly Binds to the −263/−239 Region of the PSME4 Promoter to Repress Its Expression During Aging Processes

To elucidate the mechanisms responsible for reduced PA200 expression as senescence proceeds, we next explore the transcriptional regulation of *PSME4*. We first cloned different regions of the *PSME4* upstream promoter (Figure S2a,b) and inserted them into the luciferase reporter plasmid pGL3, thereby constructing a luciferase reporter system containing seven promoter fragments of varying lengths (Figure S2c). Renilla luciferase, which served as an internal control, was cotransfected with these constructs into HepG2 cells. The results revealed that the region upstream of −74 was essential for *PSME4* transcription (Figure S2d). To narrow the exact regulatory regions, we predicted potential transcription factor-binding sites within this promoter using MatInspector and identified a putative p53 binding site spanning the −263 to −239 region, which contains two conserved p53 binding sequences [31,32,33] (Figure 3a). These findings suggested a potential regulatory interaction between p53 and *PSME4*.

Furthermore, p53 overexpression specifically inhibited the luciferase activity of the *PSME4* promoter fragment spanning −500 to +138 but not that of the fragment spanning −133 to +138 (Figure 3b). Consistent with these findings, transfection of p53 significantly suppressed *PSME4* transcription in a dose-dependent manner (Figure 3c–e and Figure S5). Conversely, silencing *TP53* with siRNA led to a marked upregulation of *PSME4* transcriptional levels (Figure 3f). In addition, compared with wild-type cells, p53 KO cells presented elevated PA200 expression, which was restored to the basal low level upon p53 complementation (Figure 3g). Moreover, treatment of the colorectal carcinoma cell line HCT116 with the p53 activator doxorubicin [34] led to a gradual decrease in PA200 expression in both a dose- and time-dependent manner (Figure 3h–j and Figure S2e). These results confirmed the negative regulatory role of p53 in *PSME4* transcription.

To further elucidate the function of p53 as a transcriptional suppressor of *PSME4*, we first performed endogenous ChIP-PCR to assess whether p53 occupies the native *PSME4* promoter. A specific PCR fragment spanning this candidate binding site was amplified from anti-p53 immunoprecipitates, whereas no signal was detected in the mouse IgG control, verifying that p53 binds to this region on the endogenous *PSME4* locus (Figure 4a). We next constructed three mutant plasmids in the pGL-luciferase system: 638-mut1, in which the CAAG motif was mutated into TAAT; 638-mut2, in which the CCTG motif was mutated to TTCA; and the dual mutant 638-dmut harboring both mutations (Figure 4b). The *PSME4* promoter was significantly increased in cells transfected with 638-mut1 and 638-dmut than in those transfected with the WT plasmid, whereas no significant change was observed in cells transfected with 638-mut2 (Figure 4c), indicating that the CAAG motif served as the key p53 binding site in the *PSME4* promoter. Consistent with these results, chromatin immunoprecipitation (ChIP) assays using an anti-p53 antibody revealed robust enrichment of the *PSME4* promoter fragment in the WT (pGL3-638) group, whereas enrichment was markedly reduced in the 638-mut1 and 638-dmut groups. In contrast, compared with the WT group, the 638-mut2 group retained strong p53 binding ability (Figure 4d,e). Additionally, in both the H_2_O_2_-induced senescent tumor cell model and the primary MEF cell senescence model, PA200 abundance exhibited a progressive decrease as p53 expression gradually increased during senescence progression (Figure 4f,g). Taken together, during cellular senescence, p53 represses *PSME4* transcription mainly via direct binding to the CAAG motif within the −263 to −239 region of the *PSME4* promoter.

### 2.3. PA200 Promotes p53 Expression by Inducing the Degradation of β-Catenin

As a crucial proteasome activator, PA200 is indispensable for maintaining intracellular protein homeostasis. Therefore, elucidating how cells counteract the inhibitory effect of p53 on *PSME4* transcription is of great biological significance. Intriguingly, we observed a reciprocal regulatory relationship between PA200 and p53. The deletion of PA200 not only dramatically suppressed p53 expression but also blunted the upregulation of p53 expression upon cellular aging (Figure 4e,f), whereas restoration of PA200 expression efficiently rescued p53 expression at both the protein and mRNA levels (Figure 5a,b). Thus, although p53 transcriptionally inhibited *PSME4* expression, PA200 in turn positively upregulated p53 expression. This reciprocal regulation formed a negative feedback loop that sustained intracellular PA200 protein homeostasis.

To investigate the regulatory mechanism through which PA200 modulates p53 expression, we first compared protein expression profiles between WT and PA200 KO MEFs. KEGG pathway enrichment analysis revealed significant enrichment of the Wnt signaling pathway (Figure S3a). Notably, β-catenin, the core effector of the Wnt pathway, has been reported to inhibit p53 activity [35]. However, whether β-catenin suppresses p53 expression during the aging process has remained unknown. Given that PA200 is well known as a proteasome-activating subunit, we hypothesized that β-catenin might be a nonhistone substrate of the PA200 proteasome. Consistently, PA200 deficiency increased β-catenin expression at the protein level but not at the transcriptional level and simultaneously inhibited β-catenin degradation (Figure 5a,b). We further treated cells with cycloheximide (CHX) to block new protein synthesis and measured β-catenin protein levels at 0, 2, and 4 h to assess its degradation rate in the absence of, or in combination with, the proteasome inhibitor MG132 to suppress proteasome-dependent protein degradation. Our results revealed that MG132 significantly blocked the degradation of β-catenin (Figure 5c). Moreover, PA200 overexpression accelerated β-catenin degradation upon treatment with CHX, whereas PA200 loss suppressed this degradative process induced by the histone deacetylase inhibitor TSA (Figure 5d,e). Consistently, Overexpressing PA200-WT, but not the BRDL-domain mutant PA200-Mut (N1716T/F1717S, within aa 1650–1738), accelerated β-catenin degradation in PA200-deficient cells (Figure 5f). We further detected the physical interaction between PA200 and β-catenin in A549 cells (Figure 5g). Collectively, these data suggest that PA200-associated proteasome-mediated β-catenin degradation is likely acetylation-dependent.

To further elucidate the upstream and downstream regulatory hierarchy between p53 and β-catenin, we employed two independent siRNAs targeting CTNNB1 for specific knockdown. Loss of PA200 expression reduced p53 levels while markedly upregulating β-catenin expression. Importantly, β-catenin knockdown effectively restored p53 abundance irrespective of PA200 status (Figure 5h), indicating that PA200-mediated β-catenin degradation played a vital role in ensuring the bidirectional regulation between PA200 and p53. We further assessed the subcellular localization and transcriptional activity of accumulated β-catenin upon PA200 loss. Immunofluorescence staining showed that total β-catenin signal was increased in PA200-KO cells, with clear accumulation observed within DAPI-stained nuclei (Figure S3b). Consistent with the robust nuclear accumulation of β-catenin, canonical TCF-LEF-dependent Wnt target genes, *AXIN2*, *MYC,* and *CCND1,* were significantly up-regulated in PA200-KO cells (Figure S3c). These data suggest that loss of PA200 drives β-catenin nuclear accumulation, which may enhance canonical Wnt-driven transcriptional responses.

In summary, insufficient PA200 hindered β-catenin degradation upon cellular senescence, potentiating β-catenin-mediated suppression of p53. The PA200-β-catenin-p53 axis established a negative feedback loop that sustained p53-PA200 cellular homeostasis.

### 2.4. PA200 Deficiency Significantly Inhibits Urethane-Induced Lung Tumorigenesis in Middle-Aged Mice

To assess the effects of PA200 on tumorigenesis during aging, we established a urethane-induced lung tumor model in young and middle-aged mice (Figure 6a). This model produces both pre-malignant adenomas and malignant adenocarcinomas and is widely used as an experimental system relevant to human NSCLC. During tumor induction, mouse body weight was monitored to further evaluate overall health. After 15 weeks of drug treatment, neither mouse body weight nor lung weight significantly differed between the groups (Figure S4a,b). PA200 deficiency significantly reduced tumor numbers in both young and middle-aged mice (Figure 6b), with a more pronounced inhibitory effect observed in middle-aged mice (Figure 6c). Notably, PA200 deletion suppressed Ki-67 expression in tumor tissues of middle-aged mice but not in those of young mice (Figure 6d), indicating that PA200 played a critical role in promoting tumorigenesis in elderly individuals. Consistent with these findings, the expression of HMGB1, a marker of inflammation and cellular damage, was reduced in middle-aged PA200 KO mice compared with middle-aged WT mice, with a *p*-value approaching 0.05 (Figure 6e). Additionally, PA200 deficiency upregulated the transcription levels of proinflammatory *Il6* and *Tnf* and downregulated the expression of the anti-inflammatory factor *Tgfb*. In middle-aged mice, differences in the levels of these cytokines between WT and PA200 KO mice were no longer statistically significant (Figure 6f–h), suggesting that these three cytokine transcripts cannot fully account for the ameliorated pro-inflammatory tumor microenvironment observed in middle-aged PA200-KO mice tumors.

Furthermore, compared with WT control mice, PA200 KO mice exhibited higher levels of β-catenin in the lung tissue of the middle-aged groups (Figure 6i). This finding was consistent with the proposed model in which PA200 acted as a proteasomal activator, targeting β-catenin for degradation; therefore, its deletion led to β-catenin accumulation. Conversely, p53 levels were lower in PA200 KO mice than in WT controls in the lung tissue of both the young and middle-aged groups (Figure 6j), further demonstrating that the accumulation of β-catenin in PA200 KO mice suppressed p53 expression. On the basis of these findings, we propose a negative feedback regulatory mechanism involving p53 and PA200. As a transcriptional repressor, p53 specifically binds to the *PSME4* promoter and inhibits its transcription. As cells age, insufficient PA200 levels weaken β-catenin degradation, thereby suppressing p53 transcription. Although the downregulation of PA200 in middle-aged mice leads to cell-intrinsic protumorigenic changes, including β-catenin accumulation and p53 downregulation, these effects are overridden by a dominant, cell-extrinsic antitumor mechanism in middle-aged mice. Specifically, PA200 deficiency partially influenced the secretion of proinflammatory factors in middle-aged mice, as evidenced by reduced HMGB1 expression and a dampened SASP response, which is a major driver of tumor progression in elderly individuals (Figure 6k). This partial suppression of the inflammatory “soil” required for tumor growth may explain why, compared with young mice, middle-aged mice with PA200 knockout exhibited significantly stronger tumor resistance.

## 3. Discussion

In this report, we investigated the previously unknown mechanism underlying the positive regulatory effect of PA200 on tumorigenesis, especially in aging individuals. This study highlights the importance of the stability of PA200 in maintaining the cell-intrinsic homeostasis of p53, as well as the cell-extrinsic tumor environment of the SASP. The present study provides a novel model in which an autoregulatory feedback loop exists between p53 and PA200. We believe that cellular homeostasis is disrupted during cell senescence, leading to the upregulated expression of p53 to exert its biological functions, such as the inhibition of cell proliferation and arrest of cell-cycle progression. p53 suppresses the transcription of *PSME4*, thereby inducing the accumulation of β-catenin, a potential non-histone substrate of the PA200-containing proteasome. In turn, β-catenin further inhibits p53 expression. Although reduced PA200 expression triggers p53 upregulation, this intrinsic compensatory effect might be overridden by the dampened SASP, which ultimately results in suppressed tumor growth.

The tumor suppressor protein p53 is essential for the maintenance of normal intracellular homeostasis. At least 50% of cancers harbor p53 mutations or functionally defective p53 proteins [36]. Cellular stresses, including DNA damage and hypoxia, induce p53 upregulation, which subsequently activates a series of downstream genes involved in DNA repair, cell cycle arrest, apoptosis, and autophagy, thereby inhibiting tumorigenesis [37,38]. As a sequence-specific transcription factor, p53 binds to specific genomic loci to modulate the transcription of its target genes [39,40]. In the present study, we identified a putative p53-binding motif, “CAAG”, within the promoter region of *PSME4* and confirmed that p53 functions as a transcriptional repressor of *PSME4*. Notably, PA200, a critical proteasome activator, reciprocally potentiates p53 expression in a β-catenin-dependent manner. In contrast, PA28γ, another proteasome activator, has been reported to facilitate MDM2-induced ubiquitination and proteasomal breakdown of p53, which blocks p53 accumulation and abrogates DNA damage-triggered apoptosis [32,41,42]. Mechanistically, Smad3 and p53 can repress PA28γ expression via the recruitment of corepressors. This regulatory cascade impedes the degradation of tumor suppressors, inhibits malignant progression and cancer cell proliferation, and alleviates the chemoresistance of tumor cells [43]. Collectively, these findings demonstrate that the two ATP- and ubiquitin-independent proteasome activators have distinct regulatory effects on p53 stability. It is highly likely that proteasome activator-mediated regulation of p53 is cell type- and cell context-specific.

Although the regulatory interplay between mutant p53 and *PSME4* transcription, as well as the modulatory effects of TGF-β/Smad signaling on PA200 during tumor progression, remains to be further elucidated, the oncogenic role of PA200 has been well established. Accumulating evidence has validated the potential function of PA200 in tumorigenesis. In hepatocellular carcinoma, PA200 facilitates tumor cell proliferation via mTOR signaling [44]. In NSCLC, PA200 increases the invasive potential of tumors, mediates immune suppression, and decreases tumor responsiveness to immune checkpoint inhibitor therapy [29,45]. Aging and malignancy are tightly interconnected through shared molecular pathways that govern both tumor initiation and age-related pathological deterioration [2]. To our knowledge, the present study is the first to elucidate the specific function of PA200 in modulating aging-driven tumorigenesis. Herein, we first demonstrate that PA200 endows senescent tumor cells with enhanced proliferative and migratory capabilities. Notably, PA200 deficiency triggers the most pronounced cell cycle arrest in senescent tumor cell populations. At the cellular level, PA200 depletion compromises β-catenin degradation during cellular senescence, thereby reinforcing the β-catenin-mediated expressional repression of p53. Consistent with our in vitro observations, a urethane-induced mouse lung cancer model further confirmed that PA200 ablation increases β-catenin levels while reducing p53 expression in vivo. Moreover, PA200 deficiency markedly suppresses tumor formation, particularly in middle-aged mice. Histological staining further confirmed that PA200 inhibits cellular senescence and promotes tumor cell proliferation. In addition, PA200 deletion substantially alleviated tumor burden in middle-aged mice. This anti-tumor effect occurs largely independent of Il-6/TNF-α-driven inflammatory responses in middle-aged hosts, although PA200-deficient tumors exhibited a non-significant trend toward lower HMGB1, suggestive of partial, age-restricted remodeling of the tumor microenvironment. These findings provide evidence that supports PA200 as a promising therapeutic target for antitumor treatment in elderly individuals.

The present study employed tiered complementary datasets and models. Pan-cancer TCGA and GEO analyses first established broad clinical associations between elevated PA200 expression and poor survival across multiple tumor types. Initial mechanistic assays were performed in HeLa, HepG2, and HCT116 cells, with HeLa serving as a general mechanistic model for proliferation, migration, senescence, and cell-cycle analyses, supporting the functional generality of the PA200-TP53-β-catenin axis across tumor backgrounds. To anchor these findings to lung cancer, the focus of our urethane-induced in vivo model, we further validated this axis in A549 and H460 lung cancer cell lines. This multi-layered design integrates pan-cancer clinical evidence, broad mechanistic characterization, and lung-cancer-specific cellular and in vivo validation to support our central hypothesis.

Our study highlights the link between p53 and the PA200 proteasome via β-catenin. It is generally believed that wild-type p53 and β-catenin share a sophisticated reciprocal regulatory network that governs tumor cell homeostasis, senescence, and malignant progression. Mechanistically, p53 serves as a critical negative regulator of β-catenin signaling. Functional p53 promotes β-catenin ubiquitination and proteasomal degradation, thereby inhibiting the oncogenic Wnt/β-catenin transcriptional activity [46,47]. Conversely, stabilized and nuclear-translocated β-catenin suppresses *TP53* transcriptional activity, forming a negative feedback loop that compromises p53-mediated cell cycle arrest and apoptotic responses [35,48]. Importantly, this crosstalk is profoundly altered during cellular senescence and aging-related tumorigenesis. In our study, PA200 deficiency was found to impair β-catenin degradation, leading to sustained β-catenin accumulation, which further represses p53 function. This disrupted balance between β-catenin and p53 may ultimately enhance the proliferative and migratory capacities of senescent tumor cells and facilitate aging-associated lung cancer progression. Consistent with our findings, multiple studies have reported that β-catenin accumulates significantly in senescent tumor cells and middle-aged tissues. Senescent cancer cells display pronounced nuclear β-catenin enrichment, which sustains oncogenic Wnt signaling and promotes malignant progression, EMT, and stemness properties [49]. Age-related impairment of the β-catenin destruction complex further stabilizes the β-catenin protein, leading to persistent β-catenin upregulation during cellular senescence [50,51]. In line with these reports, we found that PA200 deficiency promotes nuclear accumulation of β-catenin under senescent conditions. This event activates canonical TCF/LEF-dependent Wnt transcription, reinforces β-catenin-dependent p53 suppression, and ultimately drives aging-related lung cancer progression. Integrating our current data with prior work [27], we propose a feed-forward regulatory model instead of simple unidirectional control. In tumor cells exposed to oxidative stress, PA200-deficiency triggers persistent G2-phase cell-cycle arrest and long-term proliferative impairment, indicating that PA200 loss initiates and exacerbates senescence. Reciprocally, senescence-activated p53 transcriptionally represses *PSME4*, further reducing PA200 protein abundance. Therefore, PA200 downregulation acts both as an upstream trigger and a component of this senescence-associated feed-forward amplification circuit, rather than merely a terminal by-product. Of note, the middle-aged PA200-KO mouse group has a limited sample size (n = 3), which should be considered when interpreting the in vivo results.

Notably, the present results raise a conceptual hypothesis that PA200 may participate in a senescence-based “one-two punch” anticancer framework in aging-related lung cancer progression, although this therapeutic strategy has not been directly tested in our study. The canonical one-two punch therapeutic paradigm consists of two sequential steps: the first step involves the induction of stable tumor cell senescence to lock malignant cells in a nonproliferative state and increase their therapeutic vulnerability, while the second step includes the elimination of senescent tumor cells and the resolution of the protumorigenic senescence-associated microenvironment to block tumor relapse and progression [18,52]. Mechanistically, PA200 depletion triggers robust cell cycle arrest and effectively induces senescence in senescent tumor cells by blocking β-catenin degradation, leading to the accumulation of β-catenin protein, and further repressing p53-mediated tumor-suppressive signaling, all of which accomplish the core goal of the first therapeutic punch. Moreover, PA200 deficiency may modulate the tumor immune microenvironment by partially reversing the protumorigenic cytokine profile in middle-aged mice. Such changes could mitigate pro-tumor immune features and sensitize aging-associated lung cancer to subsequent antitumor interventions, thereby supporting the hypothetical second punch. Comprehensive immune-cell profiling will be required in future work to systematically characterize how PA200 shapes the tumor immune microenvironment. In summary, PA200 acts as a critical regulator that links cellular senescence, β-catenin/p53 signaling, and tumor immune microenvironment remodeling. Further pre-clinical studies are needed to evaluate whether targeting PA200 can be exploited for combinatorial anti-tumor interventions in elderly patients with lung cancer.

## 4. Methods and Materials

### 4.1. Animals

PA200 knockout C57BL/6N mice were generated using the CRISPR-Cas9 genome editing system by Beijing Biocytogen Co., Ltd. (Beijing, China), as detailed in our previous study [27]. All animal experiments were conducted in compliance with institutional animal care guidelines. Animal housing, surgical operations, and experimental manipulations were performed following the regulations issued by the Ministry of Science and Technology of the People’s Republic of China ([2006] No. 398), with prior ethical approval obtained from the Ethics and Animal Welfare Committee, College of Life Science, Beijing Normal University. Twelve-month-old C57BL/6N mice were selected as the “middle-aged” group based on our previous work demonstrating that PA200-deficient mice exhibit multiple aging-related deteriorations by 12 months of age [27]. Of note, these 12-month-old animals represent a middle-aged rather than advanced geriatric mouse model.

### 4.2. Strains and Cell Culture

The HeLa (human cervical carcinoma, *TP53* wild-type, ATCC CCL-2), HCT116 (human colorectal carcinoma, *TP53* wild-type, ATCC CCL-247), HepG2 (human hepatocellular carcinoma, *TP53* wild-type, ATCC HB-8065), A549 (human lung adenocarcinoma, *TP53* wild-type, ATCC CRM-CCL-185) and H460 (human large-cell lung carcinoma, *TP53* wild-type, ATCC HTB-177) were cultured in Dulbecco’s modified Eagle’s medium (DMEM, MeilunBio, MA0212, Dalian, China) supplemented with 10% fetal bovine serum (FBS, TIANHANG, 13011-8611, Huzhou, China), 100 U/mL penicillin, and 100 μg/mL streptomycin (Cytiva, SV30010, Shanghai, China) (for the MEFs, 1% nonessential amino acids and 200 μM β-mercaptoethanol were added). All the cells were cultured at 37 °C in a 5% CO_2_ environment. Primary PA200^+/+^ and PA200^−/−^ MEFs were isolated from the embryos of wild-type, PA200 deficient mice and used for cellular aging models. Permanent wild-type and PA200 deficient MEFs (*TP53* wild-type) were obtained from mice as previously described [25]. p53-KO HCT116 cells were kindly provided by Prof. Junjie Zhang. All cells used in this study were mycoplasma-negative.

### 4.3. Plasmid Construction

A series of truncated variants of the *PSME4* promoter, including −1970/+138, −1411/+138, −953/+138, −500/+138, −133/+138, −97/+138, and −74/+138, were cloned via PCR. The pGL3-Basic plasmid was digested with KpnI(NEB, R3142V) and HindIII (NEB, R3104V) to yield the following plasmids: pGL3-2108, pGL3-1509, pGL3-1071, pGL3-638, pGL3-271, pGL3-235, and pGL3-212. Point mutation plasmids were constructed using PCR-mediated mutagenesis. All the primers used are listed in Supplementary Table S1.

### 4.4. Immunoblotting

Cells were lysed in buffer containing 20 mM Tris-HCl (pH 8.0), 100 mM KCl, 0.2% Nonidet P-40, 10% glycerol, 1 mM ZnCl_2_, 10 mM β-glycerophosphate, 5 mM tetrasodium pyrophosphate, 1 mM NaF, 1 mM Na_3_VO_4_, and a mixture of protease inhibitors. Protein samples were separated by SDS-PAGE and transferred onto PVDF membranes. Antibodies or antisera against PA200 (Abcam, ab5620, 1:1000, Cambridge, UK), p53 (Abcam, ab1101,1:1000), β-catenin (BD, #610154, 1:1000, Franklin Lakes, NJ, USA), p16INK4a (Proteintech, 10883-1-AP, 1:2000, Rosemont, IL, USA), p21 (BD, #556430, 1:1000), and β-actin (Sigma-Aldrich, #A5441, 1:3000, St. Louis, MO, USA) were used as primary antibodies to detect the corresponding proteins. Peroxidase-conjugated anti-mouse IgG (ZSGB-BIO, ZB-2305, 1:5000, Beijing, China) or anti-rabbit IgG (ZSGB-BIO, ZB-5301, 1:5000) was used as the secondary antibody. The protein bands were visualized using an ECL detection system (Millipore, WBULS0500, Burlington, MA, USA).

### 4.5. Co-Immunoprecipitation (Co-IP)

A549 cells were lysed in ice-cold RIPA buffer (150 mM NaCl, 50 mM Tris, pH 8.0, 0.1% SDS, 0.5% deoxycholate, 0.1% NP-40 (Yeason, 20103ES60, Shanghai, China), 1 mM PMSF (Solarbio, P0100, Beijing, China), protease inhibitor cocktails, Roche, 04693159001, Basel, Switzerland). Then the cell lysates were incubated with the control mIgG, anti-PA200 antibody (2 μg per sample) overnight at 4 °C. The immunoprecipitation was carried out using protein G sepharose beads (Cytiva, GE17-0618-01). The cell lysates and precipitates were analyzed by WB using ECL detection system (Millipore, WBULS0500).

### 4.6. Quantitative Real-Time PCR (qRT-PCR)

The total RNA extracted from cells was treated with TRIZOL (Takara, 9108, Kusatsu, Japan), chloroform, isopropanol, and ethanol. In total, 2 μg of total RNA was reverse-transcribed in a total volume of 20 μL. For qRT-PCR analysis, reverse-transcribed cDNA was subjected to qRT-PCR with SYBR Select Master Mix (Thermo Fisher Scientific, A46012, Waltham, MA, USA) according to the manufacturer’s instructions and analyzed on an ABI 7500 Real-Time PCR machine (Thermo Fisher Scientific, Waltham, MA, USA). All the primers used are listed in Supplementary Table S2.

### 4.7. Cell Migration

Cells were digested with 0.25% trypsin (Thermo Fisher Scientific, 25200072) and resuspended in serum-free DMEM. A total of 800 μL of serum-containing DMEM was added to the lower chamber of each well in the transwell, and 3 × 10^4^ cells in 200 μL of medium were added to the upper chamber and cultured in a CO_2_ incubator (Thermo Fisher Scientific, Waltham, MA, USA) for 24 h. The cells in the lower chamber were fixed with 4% PFA (Aladdin, C104190, Shanghai, China) for 15 min. Subsequently, the lower chamber cells were stained with crystal violet and photographed for observation.

### 4.8. FACS Detection of the Cell Cycle

The cells were digested with trypsin, then washed with PBS and fixed with pre-chilled 70% ethanol overnight at 4 °C. The cells were stained with PI (Beyotime, C1008S, Shanghai, China) for 30 min at 37 °C, and the fluorescence was measured via flow cytometry (FACSAria™ III, Franklin Lakes, NJ, USA).

### 4.9. Senescence-Associated β-Galactosidase (SA-β-Gal) Staining Assay

The SA-β-gal assay was performed using a cell senescence staining kit (Sigma-Aldrich, CS0030). In brief, cells were seeded into a six-well plate at a density of 3 × 10^5^ cells per well and cultured for 24 h. The cells were then washed with PBS, fixed for 7 min, and incubated in 1 mg/mL X-gal overnight at 37 °C, after which positive cells were observed. At least 100 cells were counted in three or more random fields of view per group to determine the proportion of SA-β-gal-positive cells.

### 4.10. Chromatin Immunoprecipitation (ChIP)–PCR

The cells were mixed with formaldehyde to a final concentration of 1% in 10 mL of cell culture medium and incubated, after which the cross-linking was terminated using glycine. Cells were collected by centrifugation, resuspended in 200 μL of SDS lysis buffer (1% SDS, 10 mM EDTA, 50 mM Tris (pH 8.0), and 1 μg/mL aprotinin, pepstatin, leupeptin, and PMSF), and the mixture was incubated on ice. After sonication, the samples were diluted with ChIP dilution buffer (0.01% SDS, 1.1% Triton X-100, 1.2 mM EDTA, 16.7 mM Tris (pH 8.0), 167 mM NaCl, and 1 μg/mL aprotinin, pepstatin, leupeptin, and PMSF). The samples were subsequently incubated sequentially with protein A + G beads and p53 antibody, and mouse IgG was used as the negative control. DNA from the samples was extracted via the PCI method to serve as a template, and PCR amplification was conducted with the following primers:

ChIP-p53 F: AATTAAATCTATAAGGAACAAGCGGGATG;

R: TGCCTCACCGGAAACACGAG.

### 4.11. Luciferase Assay

The cultures were subsequently harvested by low-speed centrifugation. Using a Promega dual-luciferase assay system (Promega, Madison, WI, USA), 10 μL of cell input (OD_600_ = 2.0) was analyzed in a 96-well plate using a Tecan Infinite f200 microplate reader (Tecan, Durham, NC, Männedorf, Switzerland).

After transfection, the cells were washed with PBS three times and lysed in luciferase cell culture lysis buffer provided with the Luciferase Assay Kit (Promega, E1531). After brief vortexing, whole-cell lysates were centrifuged at 13,000× *g* for 2 min in the cold. The supernatant was collected in a fresh tube, and 20 μL was added to the luciferase assay substrate (100 μL). The luminescence was measured as relative light units.

### 4.12. Urethane-Induced Lung Carcinogenesis Model

2 months and 12 months, WT and PA200 KO mice were intraperitoneally injected with Urethane (0.8 mg/g, Sigma, U2500, St. Louis, MO, USA) dissolved in saline twice a week for 5 weeks. The mice were sacrificed 15 weeks after the first urethane injection. The lungs, spleen, and blood were harvested. The right lung lobes were mechanically cut into small pieces, after which the mRNA levels of related genes were detected. The left lung lobe was fixed with 4% PFA for 24 h, dehydrated, and paraffin embedded for histological analysis.

### 4.13. Xenograft Tumorigenicity Analysis

The Lewis lung cancer cell line (LLC) was cultured to the logarithmic growth stage and washed twice with PBS to prepare a cell suspension with a concentration of 1 × 10^7^ cells/mL. Each C57BL/6 mouse was injected subcutaneously with 0.2 mL of the cell suspension containing 2 × 10^6^ cells in the axilla of the right forelimb. The tumor volume was measured every 2 days, and the volume was calculated.

### 4.14. Tissue Collection and Immunostaining

Lungs were fixed in 4% paraformaldehyde at 4 °C overnight, dehydrated, embedded in wax, and sectioned at a thickness of 3 μm. The sections were deparaffinized, rehydrated, and subjected to antigen retrieval in 10 mM sodium citrate buffer. Afterwards, the sections were blocked with goat serum supplemented with 0.3% Triton X-100, incubated with antibodies against HMGB1 (Cell Signaling Technology, #6980S, Danvers, MA, USA) and Ki-67 (Abcam, ab15580), and then detected by IHC (ZSGB-BIO). The positive cells were then visualized using 3,3′-diaminobenzidine tetrahydrochloride (DAB, Proteintech, PR30018). After counterstaining with nuclear haematoxylin (MCE, HY-N0116, Monmouth Junction, NJ, USA), images were captured under a microscope (CARL ZEISS (Shanghai), Shanghai, China).

### 4.15. H&E Staining

Lungs were excised and fixed in 4% paraformaldehyde overnight, sectioned at a thickness of 5 μm, and stained with haematoxylin and eosin (MCE, HY-D0505). The cytoplasm was stained with eosin (red), and the nucleus was stained with haematoxylin (blue).

### 4.16. Pan-Cancer Gene Expression Analysis

*PSME4* (Ensembl gene ID: ENSG00000130592) transcriptional profiles across human cancers were obtained from the GEPIA database, which integrates TCGA tumor/adjacent-normal tissues with GTEx healthy tissues to enable robust tumor-normal comparisons. Raw expression values were computed as transcripts per million (TPM) and log_2_-transformed as log_2_(TPM + 1) for statistical analysis and visualization. Quality control and batch-effect correction were performed prior to downstream comparison. Detailed sample numbers for each cancer type are summarized in Table S3. Student’s *t*-test was applied for tumor-normal expression comparison, log-rank test for survival analysis, and one-way ANOVA for age-group comparative analysis. Exact *p*-values are presented in the corresponding figures and figure legends.

### 4.17. GEO Dataset-Based Age-Stratified Expression Analysis

Candidate lung cancer transcriptomic datasets were retrieved from the Gene Expression Omnibus (GEO) database by lung-cancer-associated keyword search. Pre-specified inclusion criteria included adequate sample size, availability of paired tumor-adjacent peritumoral tissues, and a broad span of patient age. GSE46539 fulfilled all criteria and was selected to evaluate age-dependent PSME4 mRNA expression in human lung cancer. In total, 67 patients were sorted by age and partitioned into three tertile subgroups (n = 23, 22, 22) to achieve balanced sample sizes across groups, generating age cut-offs at 59 years and 67 years. This tertile-driven grouping avoids imbalanced sample distribution that would occur with conventional fixed age thresholds, and minimizes the influence of extreme individual samples. One-way ANOVA was used for statistical comparison among the three age subgroups.

### 4.18. Statistical Analysis

Unless stated otherwise, an unpaired two-tailed Student’s *t*-test was used for comparisons between two independent groups. One-way ANOVA followed by post hoc Tukey’s multiple-comparisons test was applied for comparisons among three or more groups. Two-way ANOVA was used to evaluate the main and interaction effects of two independent variables. All quantitative data are presented as the mean ± SEM. Biological replicate numbers (n) for each group are indicated in corresponding figure legends. Significance thresholds were defined as * *p* < 0.05; ** *p* < 0.01; *** *p* < 0.001. Statistical analyses were performed assuming normal data distribution. Images were randomly selected under blinded conditions, and quantitative analysis was performed using ImageJ 1.54g.

## 5. Conclusions

This study demonstrates that the proteasome activator PA200 plays a critical role in aging-related lung tumorigenesis by sustaining the reciprocal β-catenin/p53 regulatory loop and maintaining protumorigenic inflammatory factor secretion. Elevated PA200 expression facilitates β-catenin proteasomal degradation, preserves p53 tumor-suppressive activity, and alleviates cellular senescence, thereby reinforcing the proliferative and migratory capacities of senescent tumor cells and driving age-dependent lung cancer progression. In contrast, genetic ablation of *PA200* stabilizes β-catenin to suppress p53 function, induces robust tumor cell senescence and cell cycle arrest, and partially modulates the SASP-mediated proinflammatory factor secretion, thereby markedly inhibiting middle-aged lung tumor growth both in vitro and in vivo. These findings identify PA200-dependent proteasomal regulation as a vital modulator of the crosstalk between cellular senescence and tumor progression, and suggest that PA200 is a promising therapeutic target for senescence-based intervention in elderly patients with lung cancer.

## Figures and Tables

**Figure 1:**
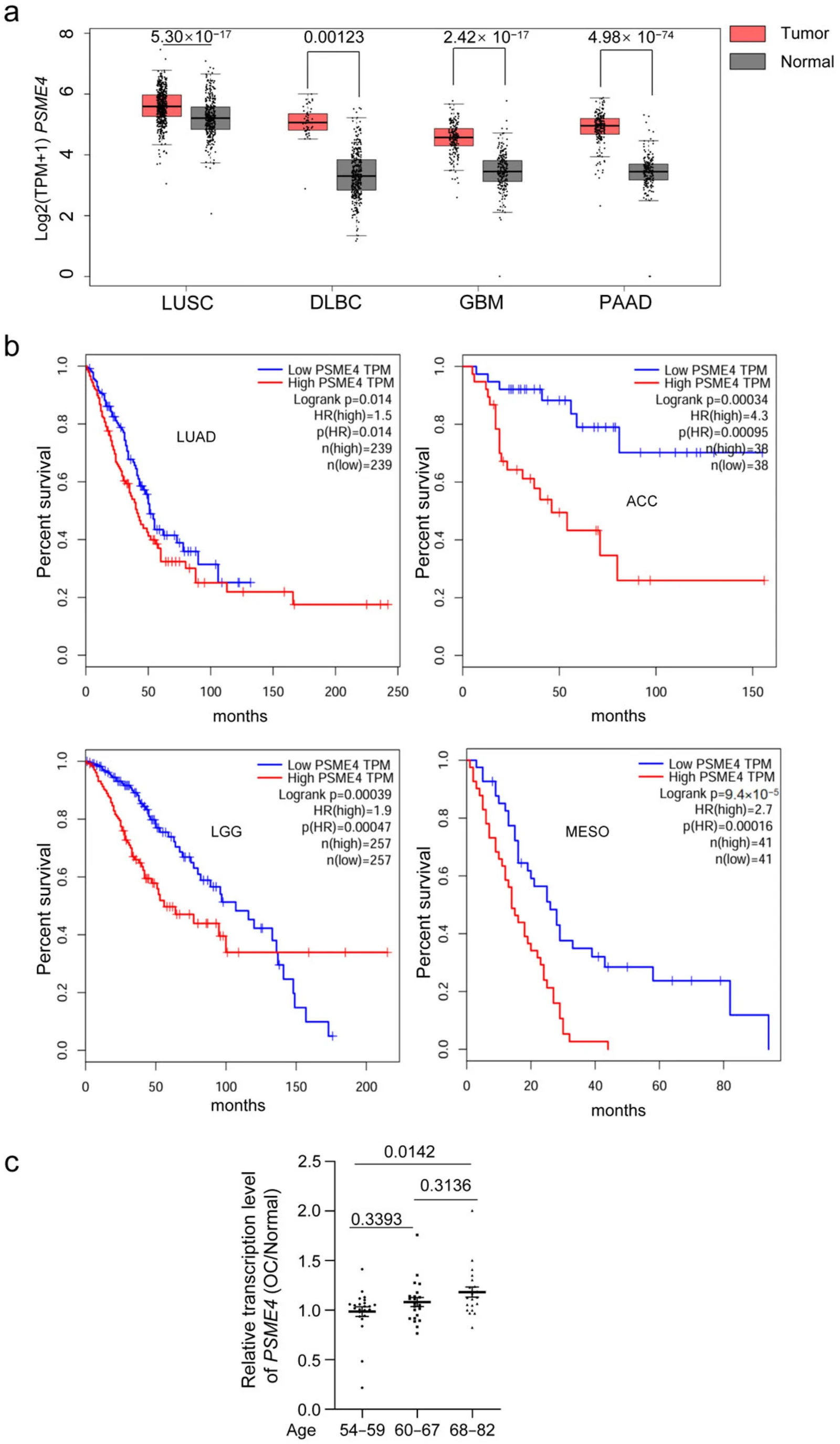
Elevated PA200 expression in human cancers is correlated with poor patient survival and advanced age. (**a**) Comparison of *PSME4* expression between tumor and normal tissues in LUSC, DLBC, GBM, and PAAD. Significance was defined as a |log2-fold change| ≥ 1 and q < 0.01. (**b**) Kaplan–Meier survival curves for PA200 in LUAD, ACC, LGG, and MESO. Patients were stratified into high- and low-expression groups on the basis of the median PA200 expression level. Survival differences were assessed using the log-rank test. (**c**) Differences in *PSME4* mRNA expression in tumor and peritumoral tissues of lung cancer patients aged 54–82 years (n1 = 23, n2 = 22, n3 = 22) from the GEO database GSE46539. Patients were sorted by age and divided into three tertile subgroups with cut-offs at 59 years and 67 years.

**Figure 2:**
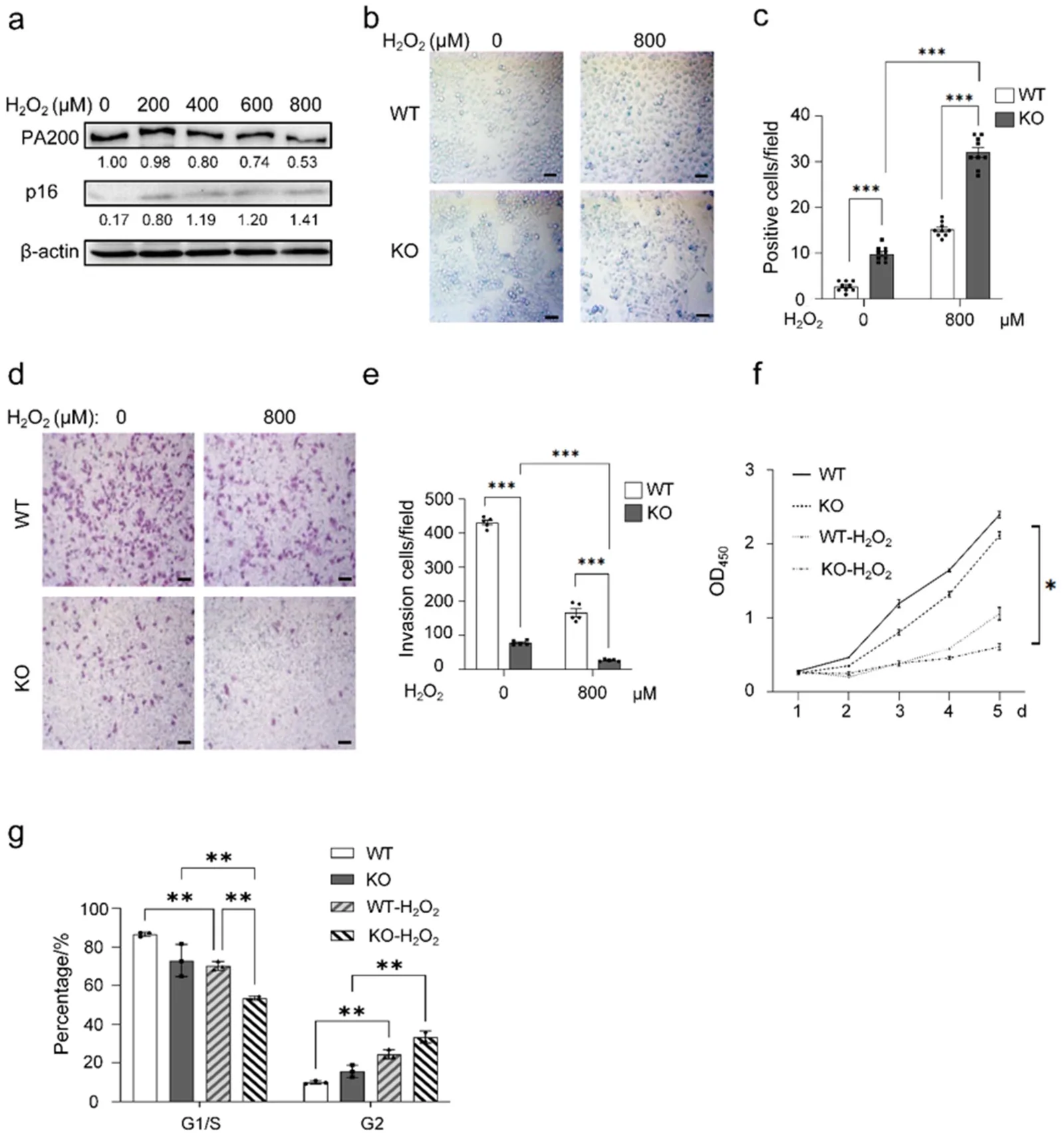
PA200 suppresses tumor cell aggressiveness under oxidative stress-induced senescence. (**a**) Immunoblot analysis of PA200 and the senescence marker p16 in WT HeLa cells treated with increasing concentrations of H_2_O_2_ (0, 200, 400, 600, 800 μM) to induce senescence. (**b**) Senescence-associated β-galactosidase (SA-β-gal) staining of WT and PA200 KO HeLa cells treated with 800 μM H_2_O_2_ for 36 h to evaluate oxidative stress-induced senescence. Scale bar, 100 μm. (**c**) Quantification of SA-β-gal–positive cells in (**b**). Data are presented as mean ± SEM from 9 fields counted per group. (**d**) The migration of cells was determined using a Boyden transwell chamber coated without Matrigel. Scale bar, 100 μm. (**e**) Quantification of migrated cells in (**d**). Data are presented as mean ± SEM from 5 fields counted per group. (**f**) Cell proliferation was assessed using a CCK-8 assay in WT and PA200 KO HeLa cells at least 3 times. (**g**) Cell cycle analysis of WT and PA200 KO HeLa cells treated with or without 800 μM H_2_O_2_ for 36 h. The cells were stained with a cell cycle staining kit and analyzed by flow cytometry. The proportions of cells in each cell cycle phase were quantified from more than 2 independent biological replicates. * *p* < 0.05; ** *p* < 0.01; *** *p* < 0.001.

**Figure 3:**
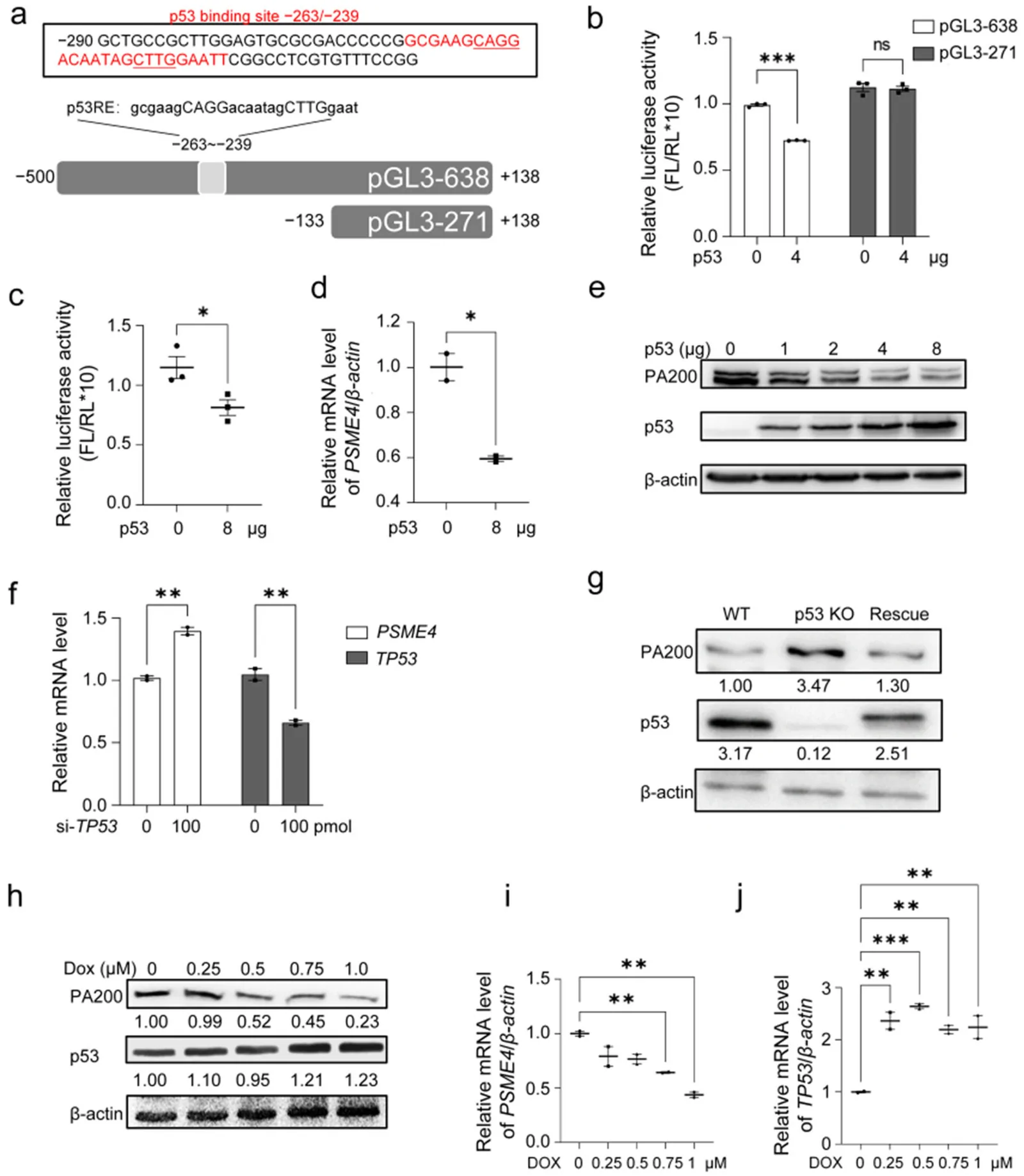
p53 inhibits the transcription of PSME4: (**a**) The p53-binding region within the *PSME4* promoter was predicted using MatInspector (**top**). DNA fragments containing or lacking this region were cloned and inserted into the pGL3 luciferase reporter plasmid (**bottom**). (**b**) Dual-luciferase reporter assay for determining the effects of p53 overexpression on *PSME4* promoter activity. HepG2 cells were co-transfected with 1 μg of *PSME4* promoter reporter plasmids (with/without the predicted p53-binding region), 4 μg of p53 expression plasmid or empty vector, and 100 ng of Renilla luciferase control plasmid. Relative luciferase activity was detected 24 h post-transfection for 3 independent biological replicates. (**c**) Luciferase reporter assay for *PSME4* promoter activity in HepG2 cells transfected with p53-expressing plasmids, n = 3. (**d**) qRT-PCR analysis of *PA200* mRNA expression in HepG2 cells treated as described in (**c**), n = 2. (**e**) Immunoblot analysis of p53 and PA200 expression in cells transfected with different doses of p53-expressing plasmids for 24 h. β-actin served as the internal control. (**f**) qRT-PCR analysis of *PSME4* and *TP53* mRNA levels in HepG2 cells transfected with *TP53*-targeting siRNA (si-*TP53*) for 24 h. The results are from 2 biological replicates. (**g**) Immunoblot analysis of PA200 and p53 expression in p53-WT, p53-KO, and p53-rescued HCT116 cells. The p53-rescued cells were generated by transfecting p53-KO cells with 4 μg of p53-expressing plasmids for 24 h. (**h**) Immunoblot analysis of PA200 and p53 expression in HCT116 cells treated with the indicated concentrations of doxorubicin for 48 h. (**i**,**j**) Quantification of *PSME4* and *TP53* mRNA levels in HCT116 cells treated as described in (**h**). Data are presented as the mean ± SEM from 2 independent biological replicates. * *p* < 0.05; ** *p* < 0.01; *** *p* < 0.001.

**Figure 4:**
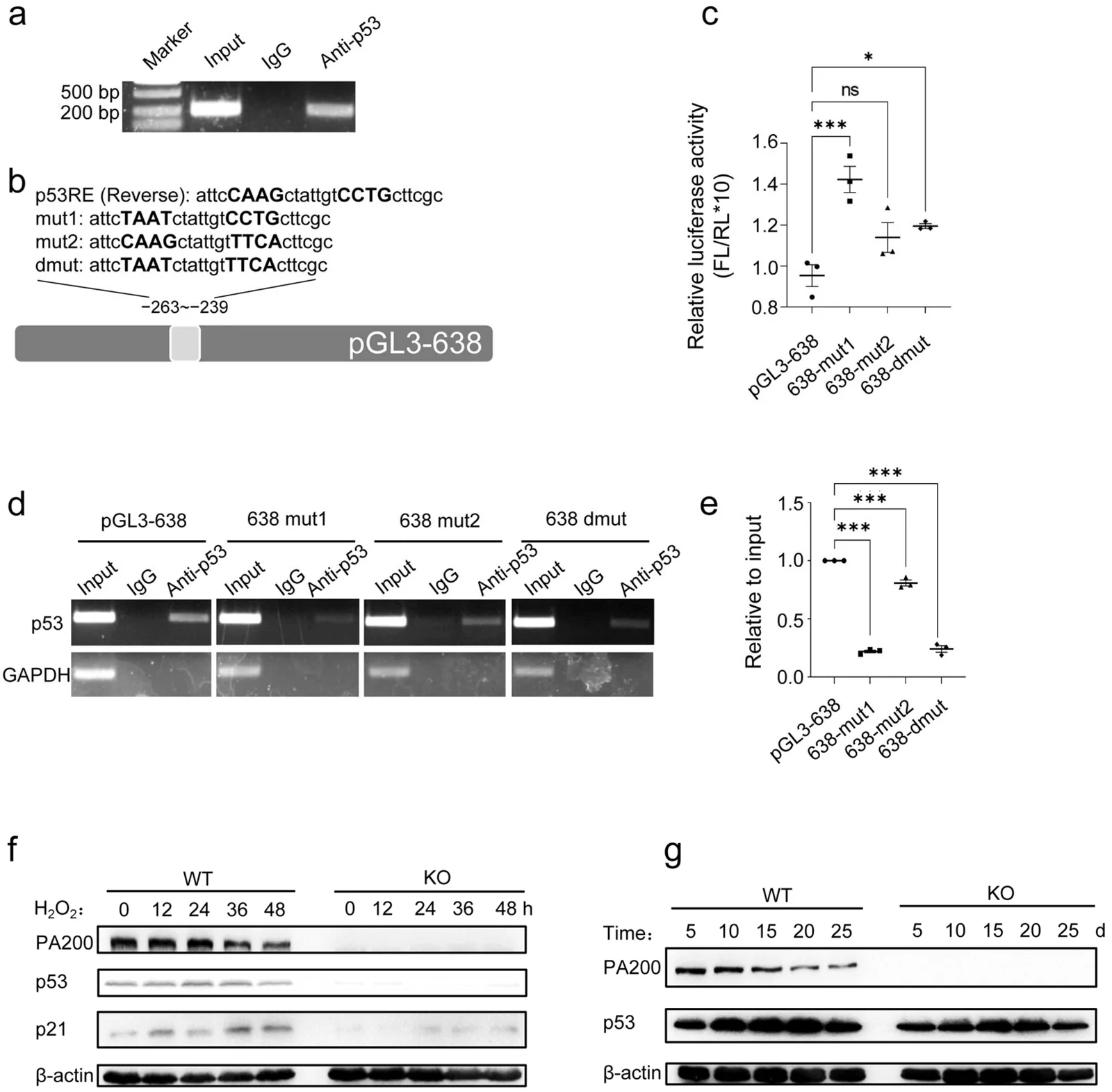
The −263/−239 region of the *PA200* promoter serves as a functional p53 binding site for transcriptional repression during cellular senescence. (**a**) Endogenous ChIP-PCR analysis verifying p53 binding to the −457/−214 region of the *PSME4* promoter in HepG2 cells. (**b**) Diagram of the pGL3-638 reporter plasmid containing wild-type or mutated p53-binding sites (−263/−239): mut1 (CAAG→TAAT), mut2 (CCTG→TTCA), and dmut (double mutation). (**c**) Dual-luciferase reporter assay in HepG2 cells co-transfected with wild-type pGL3-638 or the indicated mutant plasmids (2 μg) and Renilla luciferase control. Relative luciferase activity was measured 24 h post-transfection from 3 replicate wells. (**d**) Chromatin immunoprecipitation (ChIP) assays in HepG2 cells transfected with wild-type pGL3-638 or the indicated mutant plasmids. (**e**) Quantification of the ChIP signals shown in (**d**) using ImageJ. Data are shown as the mean ± SEM from 3 biological replicates. (**f**) Immunoblot analysis of PA200, p53, and p21 in WT and PA200 KO HeLa cells treated with 800 μM H_2_O_2_ for the indicated time. (**g**) Immunoblot analysis of PA200 and p53 in primary PA200 WT and KO MEFs during replicative senescence. * *p* < 0.05; *** *p* < 0.001.

**Figure 5:**
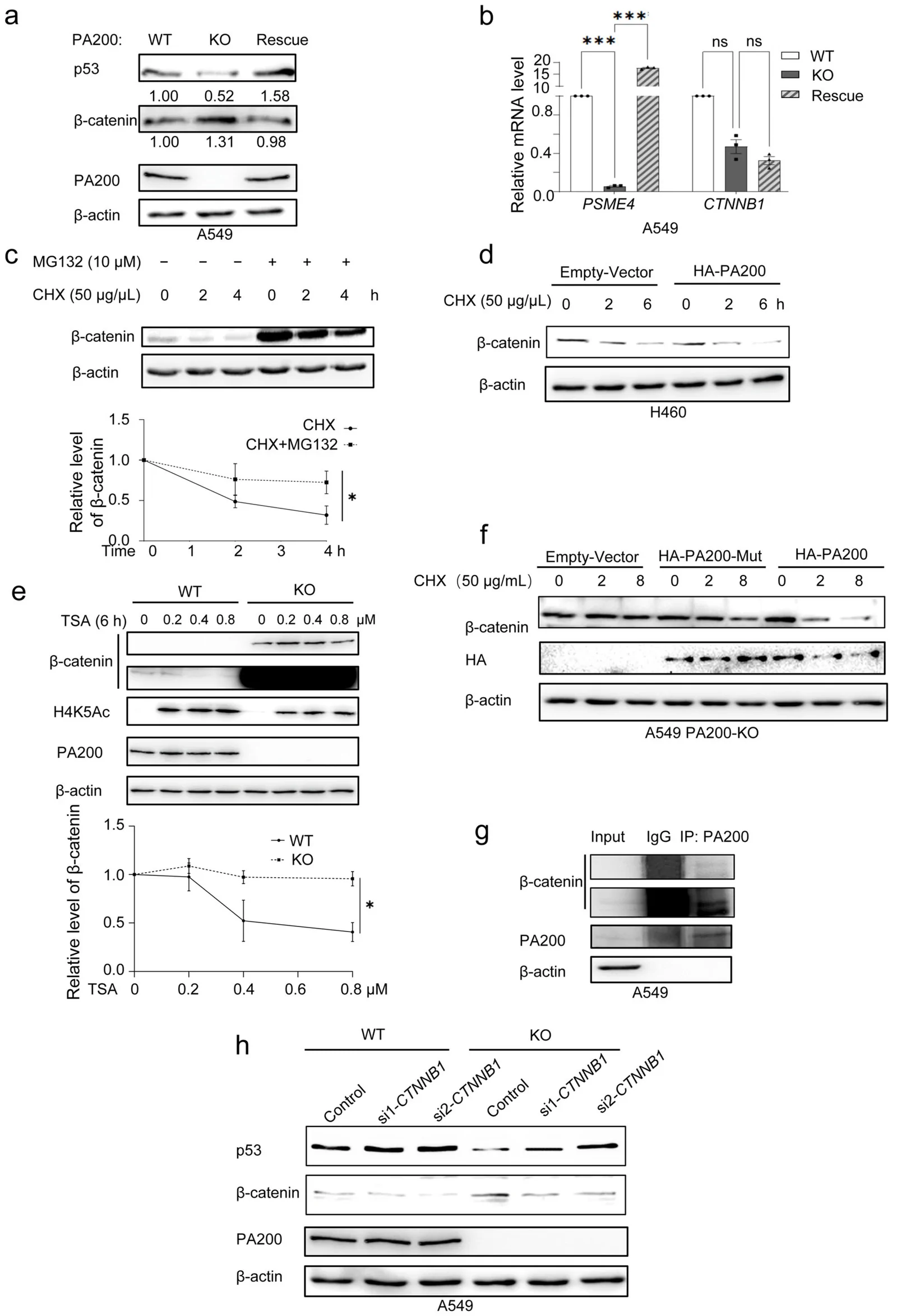
PA200 promotes β-catenin degradation, thereby increasing p53 expression. (**a**,**b**) Immunoblot (**a**) and qRT-PCR (**b**) analysis of β-catenin, p53, and PA200 in PA200-WT, -KO, and -rescued A549 cells. β-actin served as the internal control. (**c**) Immunoblot analysis of β-catenin in WT HeLa cells treated with CHX alone or in combination with the proteasome inhibitor MG132 for the indicated times. The band intensities were measured using ImageJ. (**d**) Immunoblot analysis of β-catenin in WT and PA200-overexpressing H460 cells treated with CHX for the indicated times. The band intensities were measured using ImageJ. (**e**) Immunoblot analysis of β-catenin in WT and PA200-KO HeLa cells treated with TSA at the indicated concentrations. (**f**) Immunoblot analysis of β-catenin degradation in PA200-KO A549 cells transfected with a construct expressing PA200 WT or a BRD-like mutant (BRDL-Mut). (**g**) Co-IP analysis of the binding between PA200 and β-catenin in A549 cells. (**h**) Immunoblot analysis of p53 and β-catenin in WT and PA200 KO A549 cells transfected with siRNA targeting *CTNNB1* (si-*CTNNB1*). * *p* < 0.05; *** *p* < 0.001.

**Figure 6:**
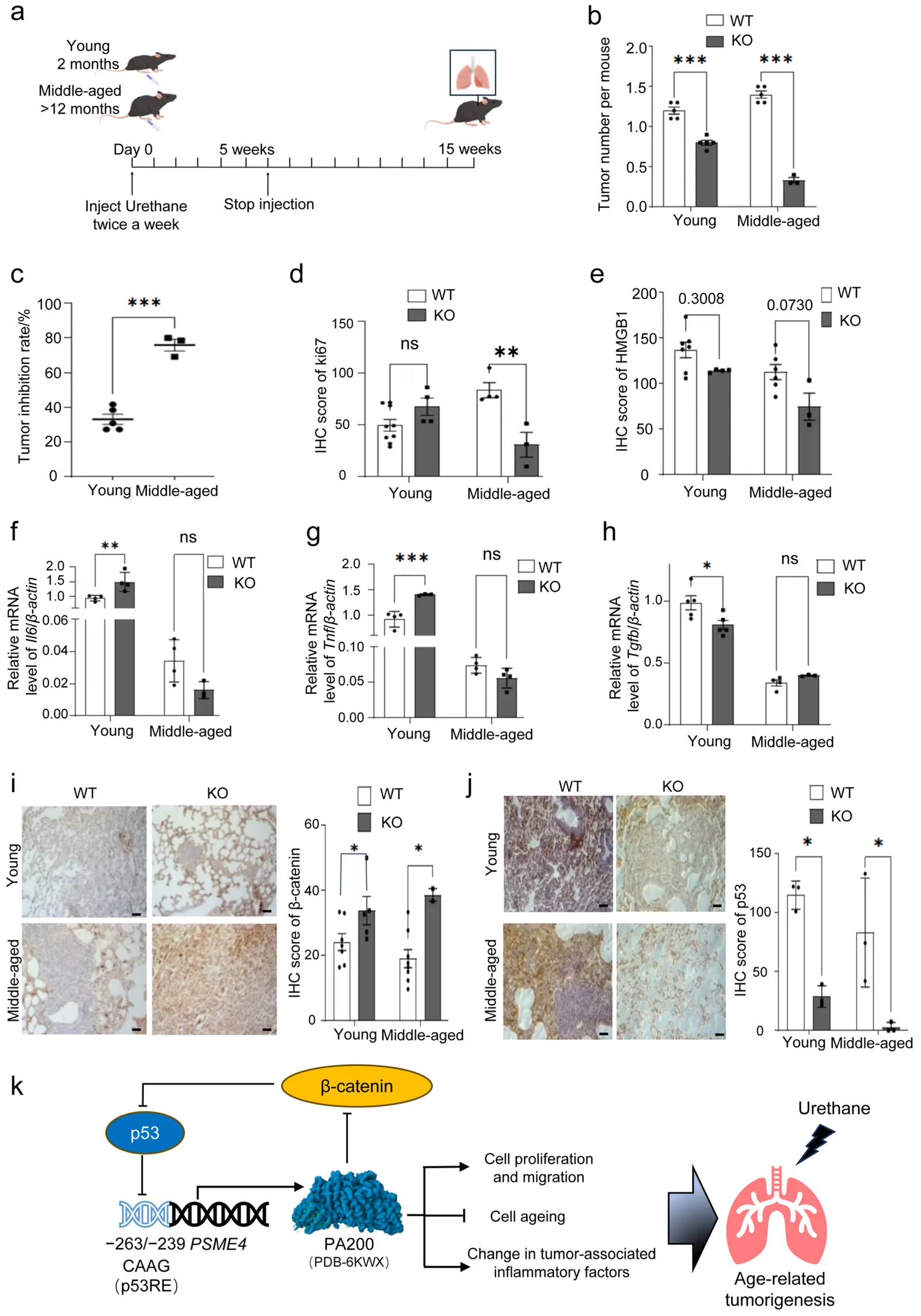
PA200 promotes urethane-induced lung tumorigenesis in middle-aged mice. (**a**) Schematic overview of the urethane-induced lung tumor model in young and middle-aged WT and PA200 KO mice. The numbers of young WT, young PA200 KO, middle-aged WT, and middle-aged PA200 KO are 5, 5, 5, and 3, respectively. (**b**) Tumor nodules per lung were quantified based on H&E-stained paraffin-embedded lung sections. (**c**) Tumor inhibition rate in young and middle-aged PA200 KO mice, as calculated from the nodule counts in (**b**). (**d**) Ki-67 staining was quantified by IHC staining of lung sections. Data are presented as mean ± SEM, n ≥ 3. (**e**) HMGB1 staining was quantified by IHC staining of lung sections. Data are presented as mean ± SEM, n ≥ 3. (**f**–**h**) qRT-PCR analysis of *Il6* (**f**), *Tnf* (**g**), and *Tgfb* (**h**) in lung tissues from urethane-treated young and middle-aged WT and PA200 KO mice. Data are presented as mean ± SEM, n ≥ 3. (**i**,**j**) IHC staining of β-catenin (**i**) and p53 (**j**) in lung sections. Scale bar, 50 μm. β-catenin or p53 staining was quantified by ImageJ. Data are presented as mean ± SEM from more than 2 fields counted per group. (**k**) Diagram of the proposed mechanism, illustrating the feedback regulation of the p53-PA200 axis and its impact on tumorigenesis and progression in aging. * *p* < 0.05; ** *p* < 0.01; *** *p* < 0.001.

## Data Availability

All the data are available in the main text or the Supplementary Materials. Data will be made available upon request.

## Supplementary Materials

The following supporting information can be downloaded at: https://www.mdpi.com/article/10.3390/ijms27198783/s1.
