# Supplementary material for: The p53-PA200 Feedback Loop Modulates Age-Dependent Tumorigenesis
Source: Int J Mol Sci. 2026 Sep 30;27(19):8783. doi: 10.3390/ijms27198783 (PMC13665431; doi:10.3390/ijms27198783)
Supplement: Supplementary file 1 [file ijms-27-08783-s001.zip › ijms-4521309-supplementary.pdf]

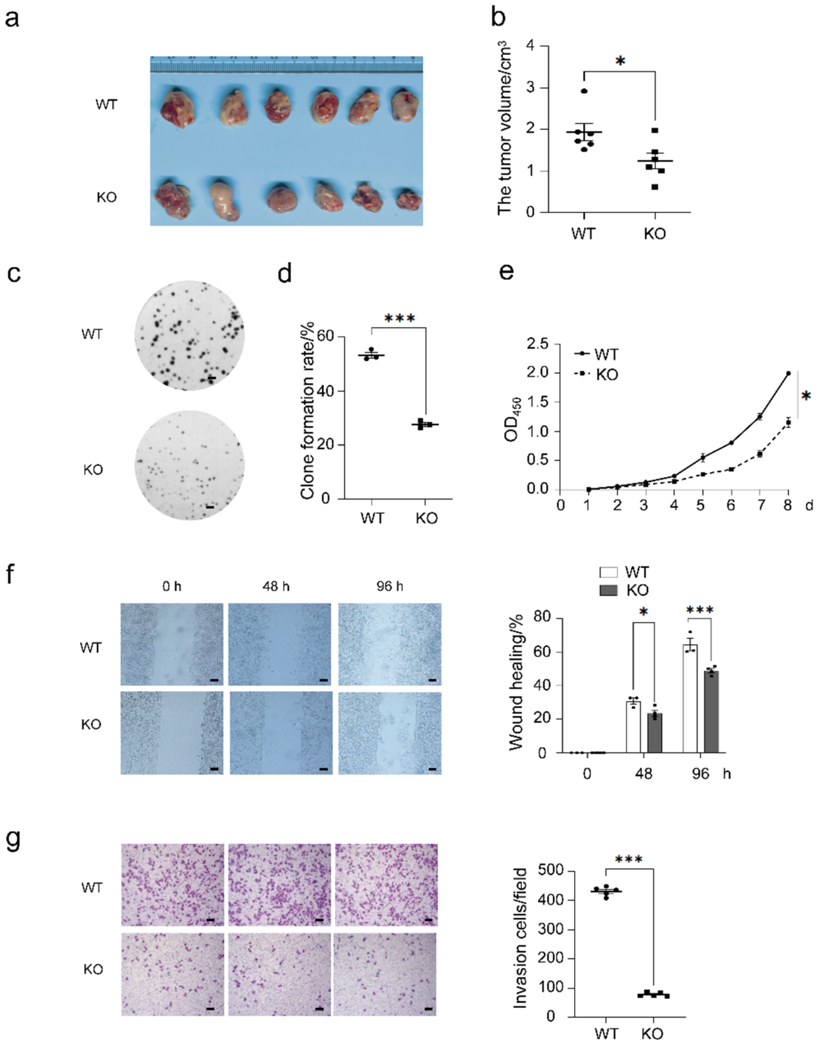

Figure S1. PA200 deficiency inhibits tumorigenesis

(a) Representative images of LLC allograft tumours in WT and PA200 KO mice. LLC cells (2×10^6) were injected subcutaneously into the axillary region of WT and PA200 KO mice. The tumours were excised two weeks later and photographed.

(b) Quantification of tumour volume from (a). The data are presented as the mean ± SEM. n=6.

(c) Colony formation assay of WT and PA200 KO HeLa cells. The cells were cultured for the indicated times, and the colonies were visualized by crystal violet staining. Scale bar, 100 µm.

(d) Quantification of colony formation from (c) for 3 independent biological repeats.

(e) The proliferation of stable knockdown PA200 cells was determined using the CCK-8 assay.

(f) Scratches were created in WT and PA200 KO HeLa cells. The wound-healing capacity was measured at 0, 48, and 96 h for 3 independent biological repeats. The wound closure area was quantified by ImageJ. Scale bar, 100 µm.

(g) The migration of stable knockdown PA200 cells was determined using a Boyden transwell chamber coated without Matrigel for 3 independent biological repeats. The left panel shows images of migration. Scale bar, 100 µm. The microscopic fields were counted for each sample. The percentage of cell migration is displayed in the right panels.

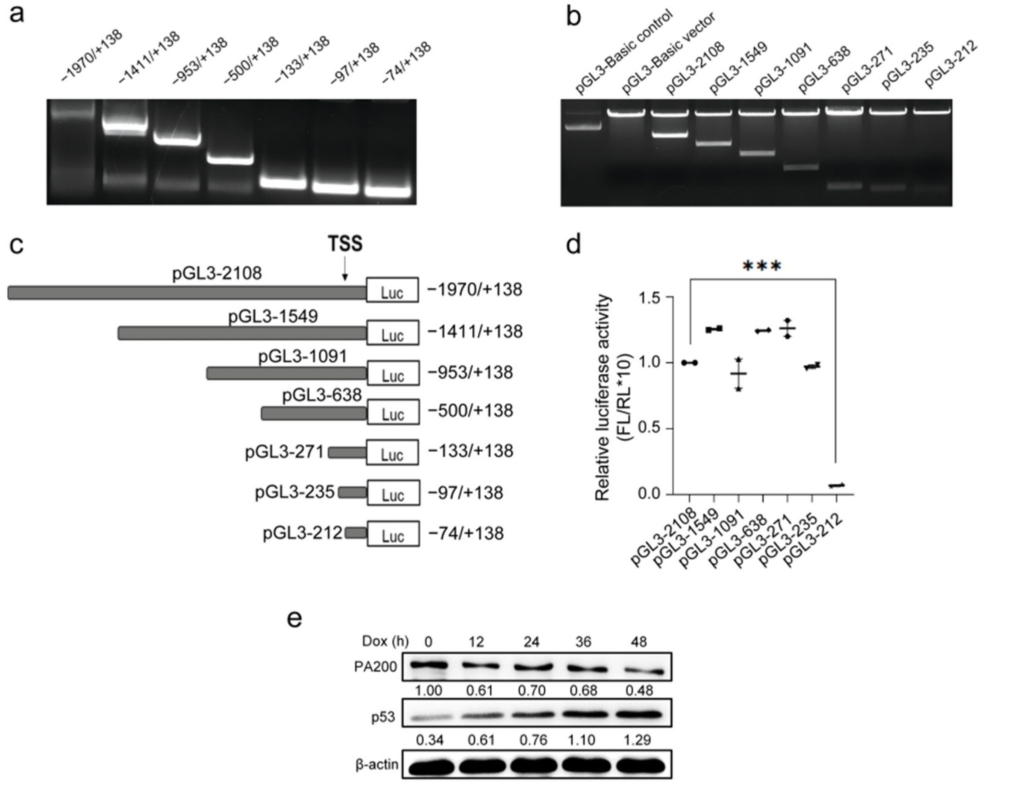

Figure S2. p53 represses *PSME4* expression at the transcriptional level during cellular ageing

(a–c) Truncated fragments of the *PSME4* promoter were cloned and inserted into the pGL3-Basic luciferase reporter vector. The recombinant plasmids were digested with KpnI and HindIII, and the products were identified.

(d) Dual-luciferase reporter assay in HepG2 cells co-transfected with the indicated *PSME4* promoter reporter plasmids and Renilla luciferase control plasmid. The relative luciferase activity was measured 24 h post-transfection for 2 repeats.

(e) Immunoblot analysis of PA200 and p53 protein levels in HCT116 cells treated with 1.0 μM doxorubicin for 0, 12, 24, 36 and 48 h.

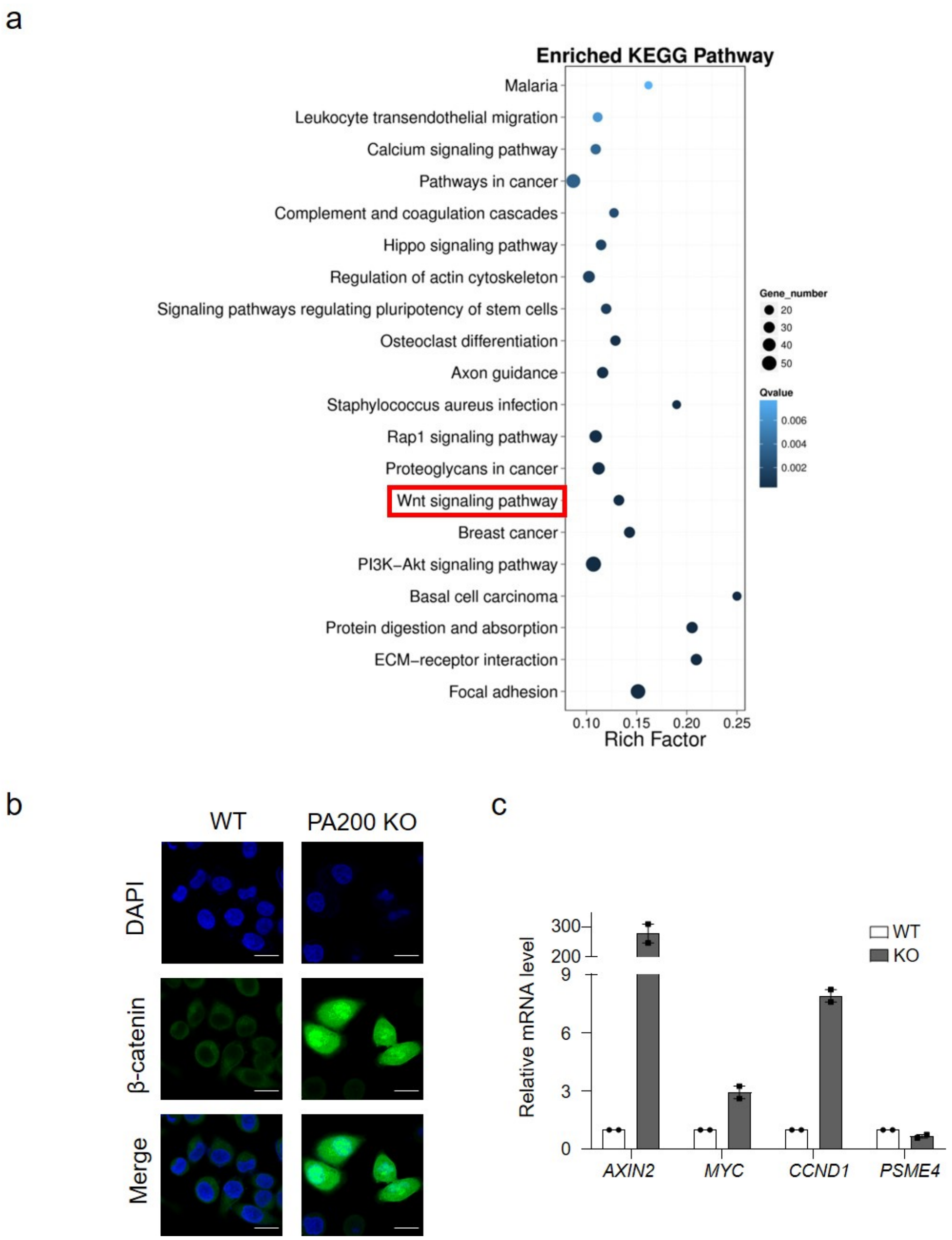

Figure S3. Wnt-related pathway enrichment and β-catenin activation upon PA200 loss.

(a) KEGG pathway enrichment analysis of differentially expressed genes (DEGs) in PA200 WT and KO MEFs.

(b) Representative immunofluorescence images showing subcellular distribution of β-catenin in control and PA200-KO HeLa cells. Increased β-catenin signal and prominent nuclear accumulation are observed in PA200-KO cells. Nuclei were counterstained with DAPI. Scale bars, 10μm.

(c) RT-qPCR quantification of β-catenin target gene transcripts (*AXIN2*, *MYC*, *CCND1*) in control and PA200-KO HeLa cells. Data are presented as mean ± SEM for 2 times.

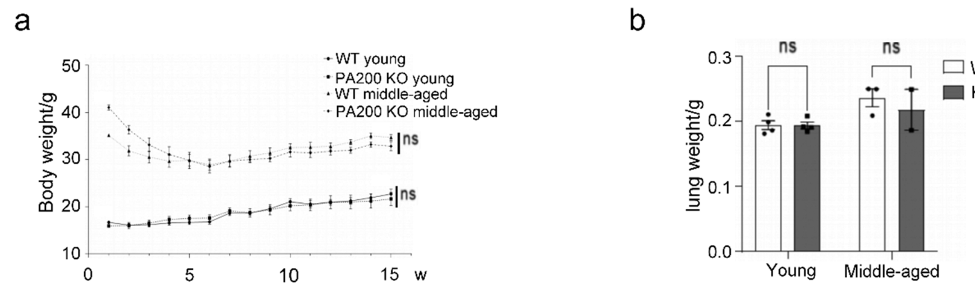

Figure S4. Body and lung weights were unaffected by PA200 KO in the urethane model.
(a-b) Weight of body (a) (n⩾3) or lung (b) (n⩾2) were detected between WT and PA200 KO mice in the urethane-induced model.

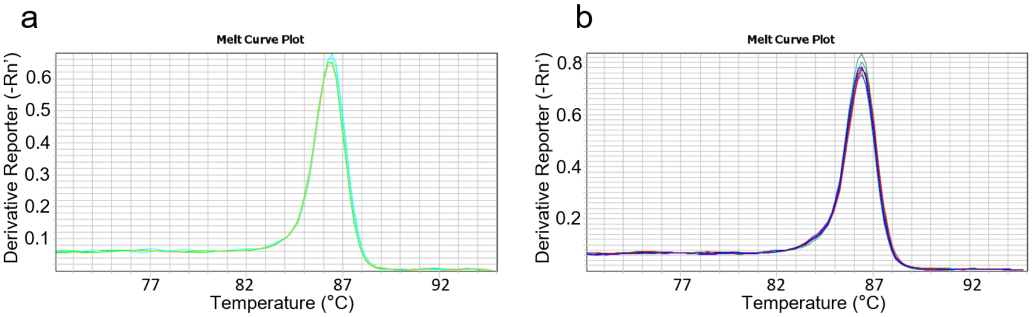

Figure S5. Melt curves of β-actin of qRT-PCR.

(a) Melt curve of β-actin in HeLa cells.

(b) Melt curve of β-actin in A549 cells.

Table S1. Primer sequences for PCR

Primers	Sequence (5'-3')
pGL3-2108	F: TTTTGGTACCTCCTTCCCTCCTGTCGTCCATCTACACGCTAATC
	R: TTTTAAGCTTACCTCCTCCGCGTCTTCGTCGCCCTG
pGL3-1509	F: TTTTGGTACCTCCATCTCCTGACCCCGTGATCCACCCG
	R: TTTTAAGCTTACCTCCTCCGCGTCTTCGTCGCCCTG
pGL3-1000	F: TTTTGGTACCAAGCTCTGAATTCCTTAGGATTGCCTTCTCAGT
	R: TTTTAAGCTTACCTCCTCCGCGTCTTCGTCGCCCTG
pGL3-638	F: TTTTGGTACCCGGCCAGAATACCAGAAAGATGGGGACTTA
	R: TTTTAAGCTTACCTCCTCCGCGTCTTCGTCGCCCTG
pGL3-271	F: TTTTGGTACCCAGCTGCCGCCACCACCGCCTCC
	R: TTTTAAGCTTACCTCCTCCGCGTCTTCGTCGCCCTG
pGL3-235	F: TTTTGGTACCCTCCGCCCCGCCCCGCC
	R: TTTTAAGCTTACCTCCTCCGCGTCTTCGTCGCCCTG
pGL3-212	F: TTTTGGTACCGTGACTTGAGGCCGGTCCGGAACCTCTG
	R: TTTTAAGCTTACCTCCTCCGCGTCTTCGTCGCCCTG
pGL3-mut1	F: GGCCGAATTCtAAtCTATTGTCCTGCTTCGCCG
	R: GGACAATAGaTTaGAATTCGGCCTCGTGTTTCCG
pGL3-mut2	F: CAAGCTATTGTttcaCTTCGCCGGGGGTCGCGCAC
	R: CCCGGCGAAGtgaaACAATAGCTTGGAATTCGGC
pGL3-dmut	F: CGAGGCCGAATTCtAAtCTATTGTttcaCTTCGCCGGG
	R: GACCCCCGGCGAAGtgaaACAATAGaTTaGAATTCGGCC
pcDNA6A-	F: ATATAAGCTTATGGAGGAGCCGCAGTCAGATCCTAGCGTCGA
p53	R: ATATGAATTCGTCTGAGTCAGGCCCTTCTGTCTTGAACATGAGT

Table S2. qRT-PCR primer sequences

Primers	Sequence (5’-3’)
H-*PSME4*	F: CCAACAGGAAAAGAATGCCGAT
	R: TAGAGACAGAAGCCCAATGCCTA
H-*TP53*	F: GGCCATCTACAAGCAGTCACAG
	R: CGCAAATTTCCTTCCACTCGGAT
H-*CTNNB1*	F: GCAGGTGGATCTATTTCATGT
	R: TGAAGCATCGTATCACAGCAG
H-*β-actin*	F: CCTGGCACCCAGCACAAT
	R: GGGCCGGACTCGTCATAC
H-*CCND1*	F: GCTGCGAAGTGGAAACCATC
	R: CCTCCTTCTGCACACATTTGAA
H-*AXIN2*	F: CAACACCAGGCGGAACGAA
	R: GCCCAATAAGGAGTGTAAGGACT
H-*MYC*	F: AGCTGCTTAGACGCTGGATT
	R: CTAACGTTGAGGGGCATCGT
M-*β-actin*	F: CATTGCTGACAGGATGCAGAAGG
	R: TGCTGGAAGGTGGACAGTGAGG
M-*Il6*	F: TAGTCCTTCCTACCCCAATTTCC
	R: TTGGTCCTTAGCCACTCCTTC
M-*Tnf*	F: TCGTATGAAATGGCAAATCG
	R: TCGTATGAAATGGCAAATCG
M-*Tgfb*	F: TGATACGCCTGAGTGGCTGTCT
	R: CACAAGAGCAGTGAGCGCTGAA

Table S3. Sample composition in GEPIA analysis

Cancer name	Number of cancer samples	Number of normal samples
LUSC	498	338
DLBC	47	107
GBM	166	110
PAAD	179	171
